# NLRP3 inflammasome deficiency attenuates metabolic disturbances involving alterations in the gut microbial profile in mice exposed to high fat diet

**DOI:** 10.1038/s41598-020-76497-1

**Published:** 2020-12-03

**Authors:** Marina Sokolova, Kuan Yang, Simen H. Hansen, Mieke C. Louwe, Martin Kummen, Johannes E. R. Hov, Ivar Sjaastad, Rolf K. Berge, Bente Halvorsen, Pål Aukrust, Arne Yndestad, Trine Ranheim

**Affiliations:** 1 Research Institute of Internal Medicine, Oslo University Hospital Rikshospitalet, Oslo, Norway; 2 Institute of Clinical Medicine, Faculty of Medicine, University of Oslo, Oslo, Norway; 3 Norwegian PSC Research Center, Department of Transplantation Medicine, Oslo University Hospital, Oslo, Norway; 4 Department of Oncology, Oslo University Hospital Ullevål, Oslo, Norway; 5 Section of Gastroenterology, Department of Transplantation Medicine, Oslo University Hospital Rikshospitalet, Oslo, Norway; 6 Institute for Experimental Medical Research, Oslo University Hospital Ullevål, Oslo, Norway; 7 KG Jebsen Center for Cardiac Research, University of Oslo, Oslo, Norway; 8 Department of Clinical Science, University of Bergen, Bergen, Norway; 9 Department of Heart Disease, Haukeland University Hospital, Bergen, Norway; 10 Section of Clinical Immunology and Infectious Diseases, Oslo University Hospital Rikshospitalet, Oslo, Norway

**Keywords:** Immunology, Cardiology

## Abstract

Obesity-related diseases (e.g. type 2 diabetes mellitus and cardiovascular disorders) represent an increasing health problem worldwide. NLRP3 inflammasome activation may underlie obesity-induced inflammation and insulin resistance, and NLRP3 deficient mice exposed to high fat diet (HFD) appear to be protected from left ventricle (LV) concentric remodeling. Herein, we investigated if these beneficial effects were associated with alterations in plasma metabolites, using metabolomic and lipidomic analysis, and gut microbiota composition, using 16S rRNA sequencing of cecum content, comparing NLRP3 deficient and wild type (WT) mice on HFD and control diet. Obese NLRP3 deficient mice had lower systemic ceramide levels, potentially resulting attenuating inflammation, altered hepatic expression of fatty acids (FA) with lower mono-saturated FA and higher polyunsaturated FA levels, potentially counteracting development of liver steatosis, downregulated myocardial energy metabolism as assessed by proteomic analyses of LV heart tissue, and different levels of bile acids as compared with WT mice. These changes were accompanied by an altered composition of gut microbiota associated with decreased systemic levels of tri-methylamine-*N*-oxide and lipopolysaccharide, potentially inducing attenuating systemic inflammation and beneficial effects on lipid metabolism. Our findings support a role of NLRP3 inflammasome in the interface between metabolic and inflammatory stress, involving an altered gut microbiota composition.

## Introduction

Obesity is a global pandemic leading to increased morbidity and mortality^1–4^. Thus, obesity-related diseases, such as insulin resistance and type 2 diabetes mellitus (T2DM), cardiovascular disorders (CVD) and non-alcoholic fatty liver disease (NAFLD) represent an increasing health problem worldwide. However, the mechanisms by which obesity contributes to these disorders are still not fully clarified^5–8^.

Inflammasomes are multiprotein inflammatory platforms that induce caspase-1 activation and subsequently release of interleukin (IL)-1β and IL-18 representing prototypical inflammatory cytokines^9^. The NOD-like receptor family pyrin domain-containing 3 (NLRP3) inflammasome responds to numerous physically and chemically diverse stimuli such as potassium efflux, reactive oxygen species (ROS), extracellular adenosine triphosphate (ATP), and various crystals including cholesterol crystals^10^. We and others have suggested that NLRP3 inflammasome may be a missing link between obesity and cardiovascular and metabolic disorders, but the molecular pathways that mediate these interactions are intensively debated and remain to be fully defined^11–14^. Studies suggest a significant role of NLRP3 inflammasome in the initiation and progression of metaflammation (i.e., metabolically-induced inflammation) and related diseases, such as obesity, T2DM, NAFLD, and atherosclerosis^15–17^. NAFLD is the most common liver disease and there are several studies suggesting that NLRP3 inflammasome, bridging inflammation and fibrosis, could play an important role and potentially representing novel targets for therapy in this disorder^18–20^. In support of this hypothesis, we have demonstrated that NLRP3 inflammasome is functional in the heart with the potential to regulate cardiac function and cell death^21^. Moreover, we have recently showed that NLRP3 deficient mice on high fat diet (HFD) were protected from adverse myocardial remodeling, and the absence of NLRP3 inflammasome components was shown to suppress obesity-induced hepatic steatosis and systemic inflammation and seemed also to have beneficial effects on glucose metabolism^22^. Long-term exposure to HFD is a relevant model for examining the effects of an unhealthy diet and obesity on the myocardium, and will reflect the situation in patients with moderate obesity, T2DM, liver steatosis and hyperlipidemia.

Several metabolic pathways may mediate the harmful effect of obesity on related cardiovascular and metabolic disorders. Thus, sphingolipids are emerging as bioactive lipids that play key roles in the regulation of cell growth, viability, differentiation, and senescence, in addition to their traditional roles in the membrane structure^23^. Moreover, bile acids are cholesterol-derived metabolites that facilitate digestion and absorption of dietary lipids. Bile acids have also emerged as pivotal signaling molecules controlling glucose, lipid, and energy metabolism, as well as inflammation^24^. Furthermore, conjugation of bile acids with glycine or taurine increases their hydrophobicity and decreases their membrane permeability, which leads to cytotoxicity, and these mechanisms have been implicated in metabolic disturbances during obesity^25^. Several studies during recent years have focused on the role of gut microbiota in mediating metabolic and inflammatory disturbances. This bacterial community in the gut plays an essential role in regulating the bile acid pool by the formation of unconjugated and secondary bile acids^26^. Moreover, through gut leakage mechanisms, bacterial products like lipopolysaccharide (LPS) may activate macrophages in abdominal fat tissue contributing to a state of metabolic induced inflammation in obese patients^26^. Indeed, changes in the intestinal microbiota have been described in obese patients and in those with T2DM and atherosclerotic disorders^27,28^. The NLRP3 inflammasome could clearly be involved in several of these processes, in particular in relation to LPS-mediated inflammation in abdominal fat tissues, but these issues are far from well understood.

The present study is a follow-up on our previous article, Sokolova et al.^22^, where we have shown that NLRP3 deficiency in mice on HFD has a beneficial effect on obesity-induced myocardial remodeling and dysfunction with attenuated infiltration of Mac-2 positive cells, insulin sensitivity, systemic inflammation (decreased levels of IL-18 and tumor necrosis factor), and liver steatosis. In this follow-up study we investigated if these beneficial effects were associated with alterations in plasma metabolites using a global metabolomic approach and gut microbiota composition, comparing NLRP3 deficient and wild type (WT) mice on HFD. For comparison we also examined corresponding changes in NLRP3 deficient and WT mice on a control diet.

## Results

Global biochemical profiles were determined in mouse plasma collected from WT and NLRP3 deficient mice fed either control diet or HFD. NLRP3 deficiency had profound effects on the plasma metabolome of mice on both control diet and HFD, and with some exceptions, the most striking effects was seen during HFD. There were particularly three categories of metabolites/pathways that were affected: complex lipids, energy metabolism, and cholesterol and bile acid metabolism.

### Complex lipids: ceramides

In WT mice HFD-induced obesity was associated with a strong increase in all of the complex lipids evaluated (i.e. phospholipids, sphingolipids, and neutral complex lipids; free FAs excluded) (Table 1). Notably, these effects were markedly less apparent in obese NLRP3 deficient mice, showing lower levels of most phospholipids, sphingolipids, and cholesterol esters as compared with obese WT mice. Interestingly, it has recently been demonstrated that obesity-related molecules, such as ceramides, can also serve as damage-associated molecular patterns (DAMPs), and are implicated in the recognition of metabolic stress and related inflammatory responses^29^. Indeed, most of the ceramides species, including short-chain and long-chain ceramides e.g. CER14:0, *P* = 0.0002; CER16:0, *P* = 0.0000; CER18:0, *P* = 0.0000; CER18:1, *P* = 0.0000; CER20:1, *P* = 0.017; CER24:0, *P* = 0.011; CER24:1, *P* = 0.0001 and CER26:1, *P* = 0.0000, were significantly increased in WT plasma on HFD as compared with levels in deficient mice. Thus, it seems that NLRP3 deficiency markedly attenuated the increase in ceramide species during obesity pointing to a role for ceramides as a mechanism for NLRP3-driven inflammation during metabolic stress.

**Table 1:** HFD resulted in strong alterations in all of the complex lipids (free fatty acids excluded) in plasma in the WT mice.

		Fold of change			
		NLRP3^−/−^		HFD	
		WT		CD	
		CD	HFD	WT	NLRP3^−/−^
**Phospholipid**	Phosphatidylcholines	1.25	*0.62*	**3.10**	**1.53**
	Lysophosphatidylcholines	1.21	*0.72*	**2.00**	1.18
	Phosphatidylethanolamines	0.95	0.72	**1.98**	**1.49**
	Lysophosphatidylethanolamines	0.99	*0.78*	**1.87**	**1.47**
	Phosphatidylinositols	1.06	0.75	**2.00**	**1.41**
**Sphingolipid**	Ceramides	1.35	*0.57*	**3.22**	1.36
	Dihydroceramides	1.09	*0.80*	**1.46**	1.07
	Hexosylceramides	**2.16**	0.63	**3.31**	0.97
	Lactosylceramides	**1.29**	0.92	**1.48**	1.05
	Sphingomyelins	**1.70**	0.63	**3.70**	1.38
**Neutral complex lipids**	Free fatty acids	0.89	0.87	1.05	1.03
	Cholesteryl esters	**1.52**	*0.66*	**2.85**	1.25
	Diacylglycerols	1.46	0.86	**1.72**	1.01
	Triacylglycerols	1.75	0.86	**2.10**	1.04
	Monoacylglycerols	1.44	0.98	**1.50**	1.02

WT and NLRP3^−/−^ male mice were exposed to high fat diet (HFD; 60 cal% fat) or control diet (CD) for 52 weeks. Table represents fold of change between NLRP3^−/−^ and WT mice on CD or HFD; and between HFD and CD in WT or NLRP3^−/−^ mice. Metabolite levels that increase in response to the diet are bold (*P* ≤ 0.05), and lipid levels that decrease are italics (*P* ≤ 0.05). [WT: CD, n = 4; HFD, n = 4 and NLRP3^−/−^: CD, n = 4; HFD, n = 4].

### Energy metabolism

#### Effects on citric acid metabolism

Obesity had little effect on plasma glucose, pyruvate, or lactate levels (Table 2). Importantly, however, NLRP3 deficient mice on control diet had higher glucose levels compared to the WT mice (*P* = 0.05), and as for lactate, NLRP3 deficient mice had higher levels independently of the diet (*P* = 0.02, CD; *P* = 0.01, HFD). This could suggest that lack of NLRP3 may increase glycolysis or the activity of lactate dehydrogenase that mediates the formation of pyruvate to lactate, or a combination thereof.

**Table 2:** Lower levels of Krebs cycle intermediates in the plasma from NLRP3^−/−^ mice on control diet.

		Fold of change			
		NLRP3^−/−^		HFD	
		WT		CD	
		CD	HFD	WT	NLRP3^−/−^
**Glycolysis, gluconeogenesis, and pyruvate metabolism**	Glucose	**1.29**	0.94	1.14	0.83
	Pyruvate	1.36	1.42	1.00	1.05
	Lactate	**1.50**	**1.57**	0.95	1.00
**Krebs cycle intermediate**	Citrate	*0.63*	0.88	0.90	**1.26**
	Aconitate	*0.63*	*0.65*	1.26	1.30
	Alpha-ketoglutarate	*0.35*	0.71	0.69	1.41
	Succinate	1.38	1.30	0.73	0.69
	Fumarate	*0.38*	1.16	*0.30*	0.89
	Malate	*0.45*	1.07	*0.36*	0.85

WT and NLRP3^−/−^ male mice were exposed to high fat diet (HFD; 60 cal% fat) or control diet (CD) for 52 weeks. Table represents fold of change in Krebs cycle intermediates between NLRP3^−/−^ and WT mice on CD or HFD; and between HFD and CD in WT or NLRP3^−/−^ mice. Metabolite levels that increase in response to the diet are bold (*P* ≤ 0.05), and lipid levels that decrease are italics (*P* ≤ 0.05). [WT: CD, n = 4; HFD, n = 4 and NLRP3^−/−^: CD, n = 4; HFD, n = 4].

Pyruvate links glucose metabolism to the Krebs cycle via the generation of acetyl CoA. Herein we found lower levels of almost all Krebs cycle intermediates in the NLRP3 deficient mice on control diet compared to WT mice on the same diet. These differences seemed to disappear when both groups were on HFD (Table 2). Thus, it appears that NLRP3 deficiency influence lactate formation and Krebs cycle metabolites with the most prominent findings during control diet.

#### Effects on fatty acid metabolism

**Figure 1:**
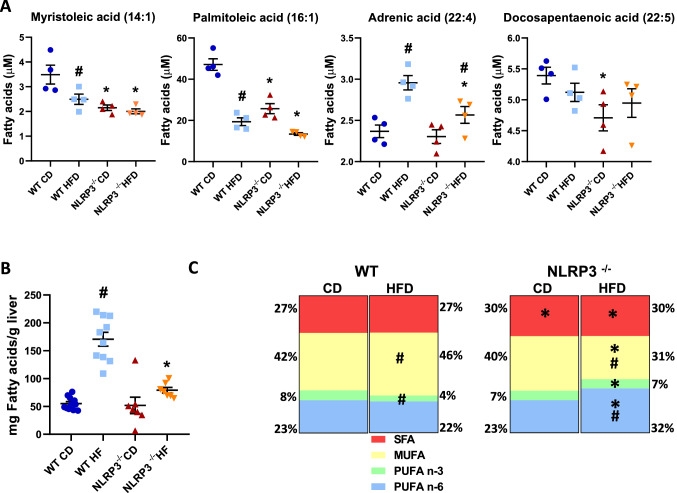
HFD resulted in higher levels of fatty acids in plasma and liver. WT and NLRP3^−/−^ male mice were exposed to high fat diet (HFD; 60 cal% fat) or control diet (CD) for 52 weeks. (**A**) Individual histograms of representative plasma fatty acids. Myristoleic acid ^#^*P* = 0.035 WT HFD vs. WT CD, *P = 0.0006 NLRP3^−/−^ CD vs. WT CD, *P = 0.024 NLRP3^−/−^ HFD vs. WT HFD; Palmitoleic acid ^#^*P* = 0.000 WT HFD vs. WT CD, *P = 0.0001 NLRP3^−/−^ CD vs. WT CD, *P = 0.003 NLRP3^−/−^ HFD vs. WT HFD; Adrenic acid ^#^*P* = 0.003 WT HFD vs. WT CD, *P = 0.04 NLRP3^−/−^ HFD vs. WT HFD, ^#^*P* = 0.048 NLRP3^−/−^ HFD vs. NLRP3^−/−^ CD; Docosapentaenoic acid *P = 0.034 NLRP3^−/−^ CD vs. WT CD. [WT: CD, n = 4; HFD, n = 4 and NLRP3^−/−^: CD, n = 4; HFD, n = 4]. (**B**) Total liver fatty acids. ^#^*P* = 0.0001 WT HFD vs. WT CD, *P = 0.0001 NLRP3^−/−^ HFD vs. WT HFD. [WT: CD, n = 11; HFD, n = 10 and NLRP3^−/−^: CD, n = 7; HFD, n = 7]. Data are means ± SEM. (**C**) Stack bars of liver fatty acid composition (weight %). SFA *P = 0.0003 NLRP3^−/−^ CD vs. WT CD, *P = 0.0001 NLRP3^−/−^ HFD vs. WT HFD; MUFA ^#^*P* = 0.008 WT HFD vs. WT CD, *P = 0.0000 NLRP3^−/−^ HFD vs. WT HFD; PUFA n-3 ^#^*P* = 0.0000 WT HFD vs. WT CD, *P = 0.0000 NLRP3^−/−^ HFD vs. WT HFD; and PUFA n-6 *P < 0.0000 NLRP3^−/−^ HFD vs. WT HFD, ^#^*P* = 0.008 NLRP3^−/−^ HFD vs. NLRP3^−/−^ CD. [WT: CD, n = 11; HFD, n = 10 and NLRP3^−/−^: CD, n = 7; HFD, n = 7]. * representing significant differences between the two genotypes (NLRP3^−/−^ and WT) fed either HFD or control diet; and # representing significant differences between HFD and control diet within one genotype, (i.e. WT or NLRP3^−/−^).

FA metabolism can also provide acetyl CoA for energy generation. Herein, the plasma levels of many medium and long chain FA, as well as polyunsaturated FAs (PUFA)s, were lower in obese NLRP3 deficient mice than in obese WT mice (Fig. 1A). These findings could be a result of decreased food intake, but this was not evident when evaluating the circulating markers of food intake from the metabolomic dataset (e.g. gluconate, 2-keto-3-deoxy-gluconate, stachydrine and homostachydrine; which are markers of food additive, viscosifier, plants and whole grain, respectively) (Supplementary Fig. S1B). In addition, the food intake was calculated in week 21, showing no difference between the genotypes (Supplementary Fig. S1A). In line with this, we have previously shown^22^, using the same model as in the present study, a significant separation of body weight between mice on HFD and control diet from week 9, determining the initial moment of obesity. Notably, although both mouse genotypes showed increased weight during HFD, WT mice gained significantly more weight than the NLRP3 inflammasome deficient mice. In contrast, no differences in weight gain between the two genotypes during control diet^22^. Furthermore, liver weights were markedly elevated in WT mice compared with NLRP3 deficient mice during HFD, but not during control diet^22^. The reduced FFA levels could also be an indication of an effect of NLRP3 on FFA synthesis or metabolism in the liver as the liver FAs in the NLRP3 deficient mice on HFD were significantly reduced compared to WT (*P* = 0.01) (Fig. 1B). Additionally, liver FA composition (weight %) showed that whereas the monounsaturated FAs (MUFAs) were decreased in NLRP3 deficient mice on HFD compared to WT mice, the PUFAs (both n-3 and n-6) were increased in NLRP3 deficient mice (Fig. 1C). Thus, whereas HFD induced a clear pattern with decreased MUFA and increased PUFA in the liver of NLRP3 deficient mice on HFD, the pattern in plasma was less obvious with a decrease in several FA even during control diet.

We also investigated immune cell infiltration in the liver by staining for Mac-2, a macrophage surface marker, and we observed a significant increase in Mac-2 positive staining in the liver in WT mice on HFD compared to NLRP3 deficient mice on the same diet (Supplementary Fig. S2A,B).

### Cholesterol and bile acid metabolism

**Figure 2:**
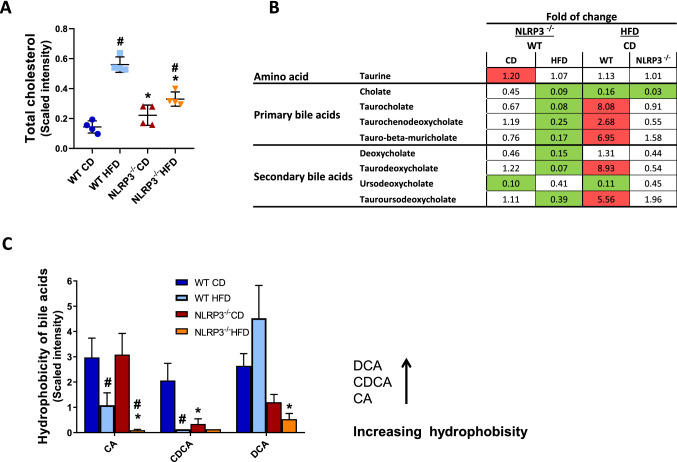
Changes in plasma cholesterol and bile acid metabolism. WT and NLRP3^−/−^ male mice were exposed to high fat diet (HFD; 60 cal% fat) or control diet (CD) for 52 weeks. (**A**) Total cholesterol ^#^*P* = 0.0001 WT HFD vs. WT CD, ^#^*P* = 0.04 NLRP3^−/−^ HFD vs. NLRP3^−/−^ CD, *P = 0.006 NLRP3^−/−^ HFD vs. WT HFD diet, *P = 0.02 NLRP3^−/−^ CD vs. WT CD. (**B**) Table represents fold of change in the amino acid taurine and primary—and secondary bile acids between NLRP3^−/−^ and WT mice on CD or HFD; and between HFD and CD in WT or NLRP3^−/−^ mice. Metabolite levels that increase in response to the diet are colored red (*P* ≤ 0.05), and lipid levels that decrease are colored green (*P* ≤ 0.05). (**C**) Hydrophobicity of bile acids. Cholate (CA) ^#^*P* = 0.01 WT HFD vs. WT CD, ^#^*P* = 0.0001 NLRP3^−/−^ HFD vs. NLRP3^−/−^ CD, *P = 0.03 NLRP3^−/−^ HFD vs. WT HFD. Chenodeoxycholate (CDCA) ^#^*P* = 0.0000 WT HFD vs. WT CD, *P = 0.0002 NLRP3^−/−^ CD vs. WT CD. Deoxycholate (DCA) *P = 0.02 NLRP3^−/−^ HFD vs. WT HFD. [WT: CD, n = 4; HFD, n = 4 and NLRP3^−/−^: CD, n = 4; HFD, n = 4]; each biochemical is rescaled to set the median equal to 1. Data are means ± SEM. * representing significant differences between the two genotypes (NLRP3^−/−^ and WT) fed either HFD or control diet; and # representing significant differences between HFD and control diet within one genotype, (i.e. WT or NLRP3^−/−^).

Cholesterol plays an important role in bile acid metabolism, and in the current study HFD-induced obesity was associated with a marked increase in total cholesterol, but importantly, this effect was significantly attenuated in obese NLRP3 deficient mice (Fig. 2A; *P* = 0.006). HFD resulted in lower levels of cholate in both genotypes, but notably, this effect was more prominent in NLRP3 deficient mice (Fig. 2B). While there was no significant difference in taurine, HFD significantly increased several taurine-conjugated bile acids (e.g. taurocholate, *P* = 0.002; taurochenodeoxycholate, *P* = 0.05; tauro-beta-muricholate, *P* = 0.01; taurodeoxycholate, *P* = 0.01; and tauroursodeoxycholate, *P* = 0.001) in the WT mice (Fig. 2B). Strikingly, these HFD-induced effects were nearly abrogated in the NLRP3 deficient mice (Fig. 2B). Interestingly, whereas there was an increase in bile acids with a higher hydrophobicity during HFD in WT mice, these bile acids decreased during HFD in NLRP3 deficient mice (Fig. 2C).

### Analysis of the left ventricular tissue during HFD

**Figure 3:**
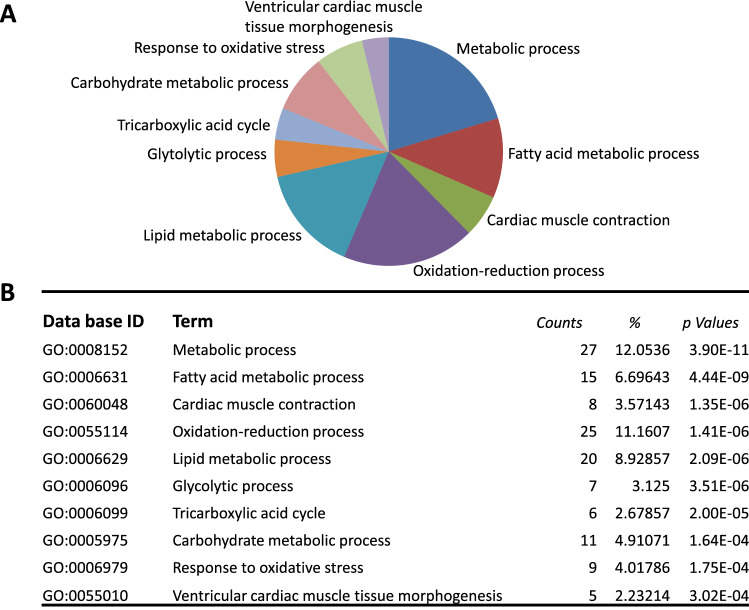
The Gene Ontology biological process categories in left ventricular (LV) tissue. WT and NLRP3^−/−^ male mice were exposed to high fat diet (HFD; 60 cal% fat) or control diet (CD) for 52 weeks. (**A**) Sector diagram of categories of the 10 most significant GO terms. (**B**) Table of the same GO terms as in (**A**). Counts reflects the number of transcripts in the pathway. [WT: CD, n = 5; HFD, n = 5 and NLRP3^−/−^: CD, n = 5; HFD, n = 5].

We have previously reported that NLRP3 deficiency had a beneficial effect on obesity-induced myocardial remodeling and dysfunction in mice^22^. To further examine the influence of HFD on myocardial remodeling in the two genotypes, we performed mass spectrometry-based proteomics of the LV. The ten most significant GO terms identified as major functional gene categories disproportionably affected by HFD in WT and NLRP3 deficient mice are illustrated in Fig. 3A,B.

**Figure 4:**
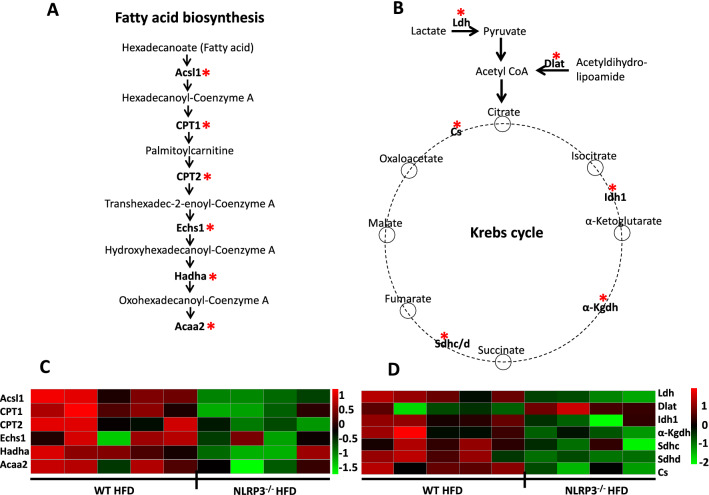
Altered transcripts in left ventricular (LV) tissue based on the KEGG pathway in the functional annotation chart of DAVID database. WT and NLRP3^−/−^ male mice were exposed to high fat diet (HFD; 60 cal% fat) or control diet for 52 weeks. (**A**) Fatty acid biosynthesis. The transcripts are differently expressed in NLRP3^−/−^ vs. WT mice on HFD. *P* = 0.0016. *Acsl1* Acyl-Coenzyme A synthase long chain family member 1, *CPT1/2* Carnitine palmitoyltransferase 1/2, *Echs1* Enoyl-Coenzyme A hydratase short chain 1, *Hadha* Hydroxyacyl-Coenzyme A dehydrogenase, *Acaa2* Acetyl-Coenzyme A acyltransferase 2. (**B**) Krebs cycle. Transcripts were significantly different regulated in the Krebs cycle in LV in WT and NLRP3^−/−^ mice on HFD. *P* = 0.0002. *Ldh* lactate dehydrogenase, *Dlat* dihydrolipoyl transacetylase, *Idh1* isocitrate dehydrogenase 1, *α-Kgdh* alpha-ketoglutarate dehydrogenase, *Sdh* succinate dehydrogenase. (**C**) Heatmap presenting differentially expressed proteins in the Fatty acid biosynthesis. (**D**) Heatmap presenting differentially expressed proteins in the Krebs cycle. Changes in protein abundance (Z-score) are shown. Proteins were grouped according to metabolic pathways. Colors represent either increased (red) or decreased (green) protein abundances. [WT: HFD, n = 5 and NLRP3^−/−^: HFD, n = 4].

In the “Fatty acid biosynthesis” pathway based on the KEGG “Fatty acid degradation” pathway, six proteins were lower in NLRP3 deficient mice on HFD compared to WT mice (*P* = 0.0016), suggesting that less FA are used as fuel for oxidation in NLRP3 deficient mice on HFD (Fig. 4A). Interestingly, six proteins involved in the Krebs cycle also showed significantly reduced levels (*P* = 0.0002), and lactate dehydrogenase was lower in the NLRP3 deficient mice on HFD (*P* = 0.0016) (Fig. 4B) indicating that NLRP3 deficiency may markedly influence the glycolytic pathway, lactate dehydrogenase activity, and the Krebs cycle intermediates also within the myocardium. Altogether, these data indicate lower overall energy metabolism in the heart in obese NLRP3 deficient mice as compared with WT mice. This altered energy metabolism within the myocardium in NLRP3 deficient mice on HFD is also illustrated by heatmaps (Fig. 4C,D). In contrast, mice fed the control diet showed no major differences in the protein pattern between the genotypes (Supplementary Fig. S4).

**Figure 5:**
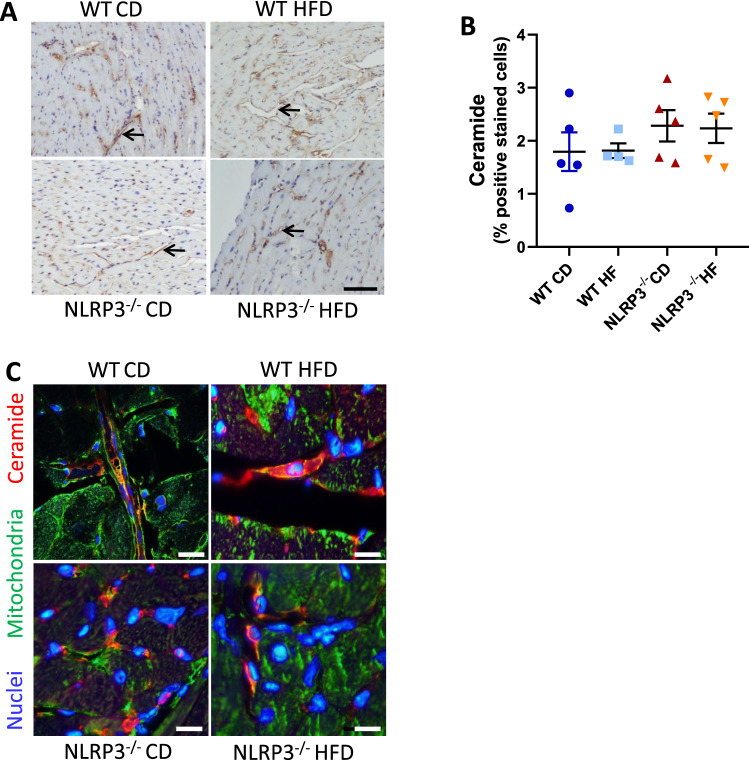
No difference in the level of ceramide in left ventricular (LV) tissue. WT and NLRP3^−/−^ male mice were exposed to high fat diet (HFD; 60 cal% fat) or control diet (CD) for 52 weeks. (**A**) Representative immunohistochemistry images of ceramide stained LV sections. Arrows are drawn to visualize ceramide staining in the endothelial cell layer. [WT: CD, n = 5; HFD, n = 5 and NLRP3^−/−^: CD, n = 5; HFD, n = 5]. Scale bar: 100 µM. (**B**) Quantification of ceramide positive stained cells. Data are means ± SEM. (**C**) Representative immunofluorescence image of LV section. [WT: CD, n = 5; HFD, n = 5 and NLRP3^−/−^: CD, n = 5; HFD, n = 5]. Ceramide in red; mitochondria marker in green; nuclei in blue; co-localization in yellow. Scale bar: 10 µM.

We additionally investigated the ceramide content in the LV tissue from the two mouse strains. Immunofluorescence showed that ceramide was co-localized in both genotypes to the mitochondria especially in the endothelial cell-layer surrounding the vessels, visualized with succinate dehydrogenase complex assembly factor (SDHAF) 2, a mitochondrial complex 2 marker (Fig. 5C). Interestingly, however, in contrast to the plasma findings, IHC revealed no difference in the levels of ceramide (Fig. 5A–C) between the genotypes. Arrows are drawn to visualize ceramide staining in the endothelial layer (Fig. 5A).

In addition to infiltrating macrophages, NLRP3 inflammasomes within the myocardium could be located in endothelial cells^30^. To test this in our model we examined the expression of NLRP3 and caspase-1 in myocardial endothelial cells in WT mice exposed to HFD or control diet (Supplementary Fig. S3). Immunofluorescence showed that the proteins were co-localized in the endothelial cells. WT mice fed a HFD showed a trend towards increased expression of NLRP3, but this difference was not statistical significant (*P* = 0.06). As for caspase-1, the localization within endothelial cells was scarce.

### Gut microbiota and systemic metabolic disturbances

**Figure 6:**
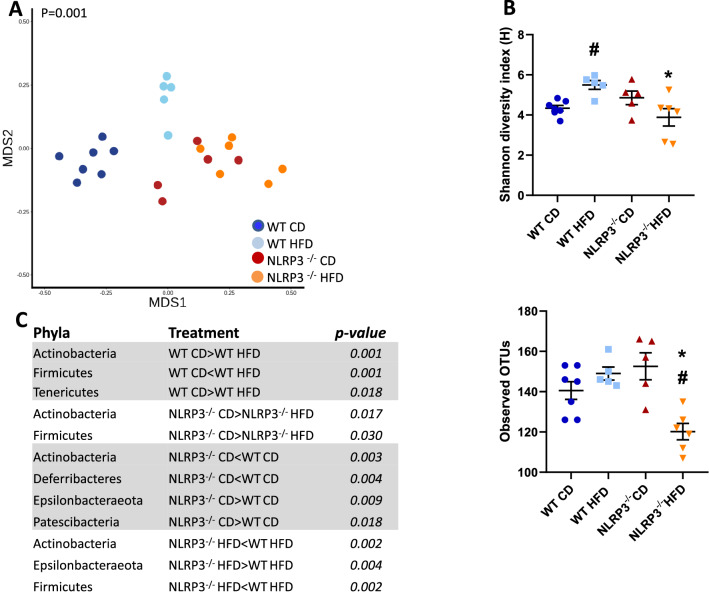
Metagenomic analysis. WT and NLRP3^−/−^ male mice were exposed to high fat diet (HFD; 60 cal% fat) or control diet (CD) for 52 weeks. (**A**) Non-metric multidimensional scaling (NMDS) plot focus on grouping sampled faecal communities with respect to diet and genotype. Datapoints represent individual mouse. The contribution of depth and region to MDS1 (primary NMDS axis) and MDS2 (secondary NMDS axis), *P* = 0.001. (**B**) Alpha diversity plots; Shannon, ^#^*P* = 0.01 WT HFD vs. WT CD, *P = 0.03 NLRP3^−/−^ HFD vs. WT HFD, and Observed OTUs, ^#^*P* = 0.008 NLRP3^−/−^ HFD vs. NLRP3^−/−^ CD, *P < 0.004 NLRP3^−/−^ HFD vs. WT HFD. (**C**) Significantly different abundances of multiple bacterial taxa at the phyla level in WT and NLRP3^−/−^ mice on CD or HFD. [WT: CD, n = 8; HFD, n = 6 and NLRP3^−/−^: CD, n = 5; HFD, n = 6]. Data are means ± SEM. * representing significant differences between the two genotypes (NLRP3^−/−^ and WT) fed either HFD or control diet; and # representing significant differences between HFD and control diet within one genotype, (i.e. WT or NLRP3^−/−^).

The gut microbiota has been suggested to influence obesity and related metabolic and inflammatory disturbances^31^. Finally, we examined the effect of diet and genotypes on the microbial profiles in the cecal content. The bacterial composition (beta diversity) was significantly different between genotypes and diets in the experimental groups (*P* = 0.001) (Fig. 6A). Moreover, the effect of diet type and genotype on microbial composition (alpha diversity) was determined using the Shannon diversity index and Observed operational taxonomic units (OTUs) for microbial composition. Whereas HFD induced increase alpha diversity in WT mice, the opposite was seen in NLRP3 deficient mice (Shannon: *P* = 0.03; OTUs: *P* = 0.004) (Fig. 6B). When exploring which taxa on the phylum levels that drove these compositional differences, we found that obese NLRP3 deficient mice had a lower relative abundance of Actinobacteria (*P* = 0.002) and Firmicutes (*P* = 0.002), and a higher relative abundance of Epsilonbacteraeota (*P* = 0.004) as compared to obese WT mice (Fig. 6C).

**Figure 7:**
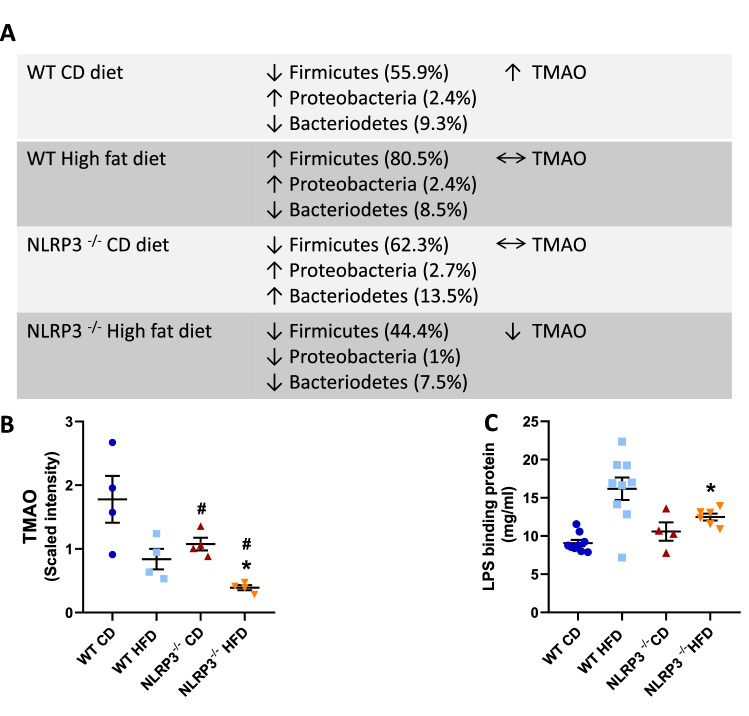
Altered bacteria profiles in gut microbiota. WT and NLRP3^−/−^ male mice were exposed to high fat diet (HFD; 60 cal% fat) or control diet (CD) for 52 weeks. (**A**) Distinct bacteria profiles in gut microbiota in WT and NLRP3^−/−^ male mice exposed to HFD or CD. Three phyla are shown; Firmicutes, Bacteriodetes and Proteobacteria. These phyla are associated to differences in levels of trimethylamine-N-oxide (TMAO). [WT: CD, n = 8; HFD, n = 6 and NLRP3^−/−^: CD, n = 5; HFD, n = 6]. (**B**) TMAO #P = 0.008 WT HFD vs. WT CD, ^#^*P* = 0.0009 NLRP3^−/−^ HFD vs. NLRP3^−/−^ CD, *P = 0.009 NLRP3^−/−^ HFD vs. WT HFD. [WT: CD, n = 4; HFD, n = 4 and NLRP3^−/−^: CD, n = 4; HFD, n = 4]; each biochemical is rescaled to set the median equal to 1. (**C**) Plasma lipopolysaccaride (LPS) binding protein. *P = 0.036 NLRP3^−/−^ HFD vs. WT HFD. [WT: CD, n = 9; HFD, n = 9 and NLRP3^−/−^: CD, n = 6; HFD, n = 4]. Data are means ± SEM. * representing significant differences between the two genotypes (NLRP3^−/−^ and WT) fed either HFD or control diet; and # representing significant differences between HFD and control diet within one genotype, (i.e. WT or NLRP3^−/−^).

Formation of trimethylamine-*N*-oxide (TMAO) is based on the microbial metabolite trimethylamine (TMA) and it has been shown that TMAO levels are associated with low abundance of Bacteroidetes and high abundance of Firmicutes, and herein we found that this pattern was seen in the WT mice on control diet and partly on HFD, but not in NLRP3 deficient mice (Fig. 7A). Indeed, NLRP3 deficient mice had lower plasma levels of TMAO compared to WT mice on HFD (*P* = 0.009) (Fig. 7B). Moreover, we found that HFD markedly increased the systemic LPS levels in WT, and notably, this effect was attenuated in NLRP3 deficient mice (*P* = 0.036) (Fig. 7C). We also showed a significant increase in Bilophila genus of the Proteobacteria phylum in obese WT mice (Supplementary Fig. S5), shown to be associated with taurin-conjugated bile acids, that was elevated in WT mice on HFD in our study (Fig. 2B)^32^. Thus, changes in gut microbiota could through attenuated levels of TMAO, LPS, and taurine conjugated bile acid have contributed to beneficial effects of NLRP3 deficiency on myocardial remodeling and liver steatosis in these mice on HFD.

## Discussion

Obesity and metabolic related cardiac disease is a growing worldwide concern^33,34^. The NLRP3 inflammasome may represent a link between overnutrition, metabolic stress, inflammation, and development of metabolic and cardiovascular diseases, but the molecular mechanisms for these interactions are not fully elucidated^35,36^. Herein we show several effects of NLRP3 deficiency on control diet, and in particular HFD associated metabolic changes in plasma, but also in the liver and within the myocardium. Thus, we show that obese NLRP3 deficient mice had lower systemic ceramide levels, a DAMP molecule with potential inflammatory effects, altered hepatic expression of FA with lower MUFA and higher PUFA levels, downregulated myocardial energy metabolism and different levels of various bile acids as compared with WT mice. Notably, these changes were accompanied by an altered composition of gut microbiota associated with decreased systemic levels of TMAO and LPS, suggesting that the decreased metabolic and inflammatory stress in NLRP3 deficient mice on HFD were related to changes in gut microbiota. We and other have suggested that NLRP3 could be a link between obesity and metabolic and inflammatory stress, and our findings herein suggest several molecular pathways that could be related to NLRP3-driven inflammation during obesity, including changes in gut microbiota.

It is becoming clear that lipotoxicity and in particular high levels of ceramides could play an important role in obesity induced inflammation^37^ as well as myocardial remodeling in obese diabetics^38,39^. Furthermore, altered sphingolipid levels, particularly ceramide and sphingomyelin, seemed to be involved in obesity-induced endothelial dysfunction and atherosclerosis^40^. Additionally, acylCoA synthase long-chain family member 1 (Acsl1) involved in ceramide synthesis and degradation^41,42^, was significantly altered in LV tissue in WT mice on HFD. Based on these studies, interest for the role of sphingolipids in obesity-induced pathobiology is emerging. In the present study we showed that NLRP3 deficiency markedly attenuated the increase in ceramide species during HFD in plasma indicating downregulation of DAMP signaling that again could influence NLRP3 activation through attenuated signal 1 activation, a priming signal indicative of infection or tissue damage^43^. However, when investigated in the LV tissue, IHC revealed no difference in the levels of ceramide between the genotypes. We have previously described the lack of both myocardial inflammation and fibrosis in our model of NLRP3 deficiency on HFD^22^ compared to others^44,45^, and it is tempting to hypothesize that this at least partly could be due to differences in cardiac ceramide accumulation. Thus, whereas our findings support a link between NLRP3-driven inflammation and ceramide signaling systemically, the role of these pathways within the myocardium could be questioned. However, we cannot exclude that NLRP3 inflammasomes modulate obesity-induced effects on the myocardium through the systemic effects of ceramides-induced inflammation. Indeed, synthesis of ceramides occurs in all tissues, and this metabolite accumulates within tissues and plasma during metabolic dysfunction, dyslipidemia, and inflammation. A number of studies carried on humans, rodents and cell cultures indicate participation of tissue and plasma ceramides in obesity and the development of insulin resistance and heart failure^46,47^. It is therefore possible that ceramides could affect the myocardium through their increased systemic levels. Alternatively, similar levels of ceramide in WT versus NLRP3^−/−^ endothelial cells might be due to a lack of local NLRP3 activation within myocardium endothelial cells. However, we found a trend for increased NLPR3 expression within myocardial endothelial cells in WT mice on HFD as compared with control diet, but these issues will have to be further investigated.

NLRP3 deficiency had several effects on energy and FA metabolism. First, plasma palmitoylcarnitine (as well as other conjugated acylcarnitines) was reduced in the NLRP3 deficient mice on both HFD and control diet compared to WT mice, and interestingly, increased levels of acylcarnitines have been observed in several metabolic and inflammatory conditions like T2DM, obesity and CVD, potentially related to mitochondrial dysfunction^48^. Second, the reduced levels of palmitoylcarnitine in NLRP3 deficient mice were also seen within the myocardium on HFD. In fact, six enzymes, including carnitine palmitoyl transferase (CPT) 1 and 2, were lower in NLRP3 deficient mice on HFD compared to WT mice, suggesting that less FA are used as fuel for oxidation in NLRP3 deficient mice on HFD with potentially beneficial effects on the myocardium^49,50^. Third, we observed significantly lower level of Krebs cycle intermediates within the myocardium in obese NLRP3 deficient mice. Recent evidence confers a new role for Krebs cycle intermediates as signaling molecules that could ameliorate activation of inflammatory pathways^51,52^, but the consequences of the disturbed levels of Krebs cycle intermediates in our model need to be further elucidated. Finally, whereas the effect of NLRP3 deficiency on the FA composition in plasma was unclear, NLRP3 deficiency on HFD show an increase in PUFA and a decrease in MUFA within the liver with potential beneficial effects on hepatic inflammation and metabolism.

Bile acids are cholesterol-derived compounds synthesized in the liver, which facilitate the intestinal absorption of lipids, but also influence metabolic and inflammatory signaling pathways^53^. In the current study, HFD induced a marked increase in total cholesterol, but importantly, this effect was significantly attenuated in the NLRP3 deficient mice. While there was no significant difference in taurine, HFD significantly increased a number of taurine-conjugated bile acids in the WT mice, but not in KO mice. Taurine-conjugated bile acids have a wide spectrum of effects and with relevance to our study. It is demonstrated that the microbiota-associated metabolites taurine shapes the host-microbiome interface by co-modulating NLRP6 inflammasome signaling, epithelial IL-18 secretion, and downstream anti-microbial peptide profiles^54^. Moreover, Jarret et al.^55^ show that neuron-derived IL-18 signaling has profound consequences on the mucosal barrier and invasive bacterial killing. In line with this, we have previously shown, using the same model as in the present study^22^, that plasma levels of IL-18 were significantly elevated in WT mice fed HFD. Given the known association between taurin-conjugated bile acids, HFD and the Bilophila genus of the Proteobacteria phylum^32^, which we found increased in obese WT mice, it is tempting to speculate that this environment could have contributed to the phenotype in these mice. Moreover, hydrophobic bile acids have been proven to affect heart rate and its contraction^56^, and have been associated with cardiac hypertrophy. It has in fact been suggested that bile acids with a higher hydrophobicity have an increased dysfunctional effect within the myocardium. Interestingly, whereas there was an increase in bile acids with a higher hydrophobicity during HFD in WT mice in our study, these bile acids decreased during HFD in NLRP3 deficient mice. Although the net effects of altered bile acid composition in NLRP3 deficient mice are at present not clear, our findings should warrant further studies on the interaction between bile acids and NLRP3 inflammasome in obesity and related disorders.

The relationship between CVD and intestinal microbiota is of great interest^57^. Cui et al. found that the composition of intestinal microbiota in patients with cardiac heart disease and healthy controls differed, particularly in the proportions of members of phyla Bacteroidetes and Firmicutes^58^. Also, the intestinal microbiota of obese mice is characterized by a higher Firmicutes/Bacteriodetes ratio when compared to lean mice^59^. Reducing levels of bacteria from the Firmicutes and Bacteroidetes phyla may improve insulin sensitivity in mice with diet-induced obesity. Herein we show that during HFD WT mice showed increased levels of Bacteroidetes and high abundance of Firmicutes, and this pattern was at least partly reversed by NLRP3 deficiency. Indeed, these changes in bacteria composition was accompanied by decreased levels of TMAO and LPS, potentially reflecting decreased levels of microbiota-derived TMA and attenuated gut leakage, respectively. Our findings further link altered gut microbiota to HFD induced inflammation. Moreover, the fact that this effect was attenuated by NLRP3 deficiency suggests that NLRP3 could be part of a missing link between the gut microbiota and systemic metabolic and inflammatory disturbances. The elevated levels of LPS, which were attenuated during NLRP3 deficiency, further support such a notion. Similarly, intestinal permeability and bacterial translocation are important contributors to chronic systemic inflammation and, might represent a continuous inflammatory stimulus capable of immune processes^60–62^ . These findings could also be relevant for patients with CVD. Elevation in plasma TMAO concentrations is associated with an increased risk of CVD in many different patient cohorts^63^. Moreover, gut leakage mechanisms with increased release of endotoxins^64^, such as the observed increase in LPS in the HFD fed WT mice, may contribute to the systemic effects of gut microbiota, potentially involving NLRP3 activation. Indeed, LPS is a potent activator of NLRP3 inflammasome (signal 1), and it is tempting to hypothesize that the interaction between gut microbiota, NLRP3 inflammasome with the induction of enhanced release of inflammatory cytokines like IL-18, LPS and TMAO could be operating in various relevant obesity-related disorders including atherosclerosis and obesity induced myocardial remodeling.

The present study has several limitations such as the lack of a positive control group by for example using MCC950, which is a potent and selective inhibitor of the NLRP3 inflammasome^65^. Furthermore, the small number of animals included in the global metabolic profiling analysis is another limitation of our study. In addition, NLRP3 inflammasomes and their components (e.g., NLRP3, caspase-1 and pro-IL-1β) should have been more convincingly located within the myocardium by western blotting. Indeed, inflammasome components are difficult to evaluate with immunofluorescence at least partly related to the transient expression of some of the binding sites for the antibodies that are used for immunofluorescence staining^66^. We also lack data from the intestinal tract which is important when studying the interaction between gut microbiota and systemic metabolic disturbances. Moreover, associations do not necessarily mean any causal relationship, and our study needs more mechanistic study to verify our findings.

To conclude, NLRP3 deficiency had profound and various effects on the plasma metabolome of mice on both control diet and in particular on HFD. These effects seem at least partly to involve altered composition of gut microbiota. Our findings further support a role of NLRP3 inflammasome in interface between metabolic and inflammatory stress, and this role seems to involve a complex interaction between fundamental metabolic pathway such as energy metabolism, FA regulation and interaction with lipid related molecules, such as ceramides and bile acids.

## Methods

### Mice

C57BL/6J mice were purchased from The Jackson Laboratory (Bar Harbor, ME, USA). *Nlrp3*^−/−^ (NLRP3 deficient) mice were generated by Millenium Pharmaceuticals (Cambridge, MA, USA), back-bred onto the C57BL/6 strain at least seven (*Nlrp3*^−/−^) generations before being used^67,68^. Mice were housed in an air-conditioned, temperature-regulated room with a 12/12 h daylight/night cycle with free access to water and food. The diet and genetic background are major determinants of gut microbial composition which again could influence metabolic and inflammatory diseases. To minimize the effects of other factors than genetics in our study, including effects on gut microbiota, the mice were co-housed throughout the study. The separate mouse strains were littermates, bred from the same parents, raised in the same cage until weaning where 4–6 mice of the same strain where co-housed in the same open cages (Eurostandard type III), and all cages were placed in the same room in a randomized manner. Obesity was induced by feeding mice a high fat diet (HFD) (D12492), composed of 60% fat, 20% protein, and 20% carbohydrate (Research Diets, New Brunswick, NJ, USA) for 52 weeks. Control mice were fed a low fat standardized control diet, containing 10% fat, 20% protein and 70% carbohydrate (D12450B, Research Diets). Body weight was monitored weekly. Food intake was determined at 21 weeks by weighing the food and correcting for the amount not eaten, including spillage. The experimental animal protocol (FOTS id 4641), was approved by The Norwegian Food and Safety Authority, which is a national governmental body that supervises food, plant, fish and animal health, and by the Norwegian Animal Research Committee that conforms to the Guide for the Care and Use of Laboratory Animals published by the US National Institutes of Health (NIH Publication, 8th Edition, 2011). All animal experiments were performed in accordance with relevant guidelines and regulations.

### Blood and tissue sampling

Mice were fasted for 4 h and put in deep anaesthesia with a mixture of 4–5% isoflurane and O_2_. Arterial blood was collected (by a small incision of the carotid artery) into tubes containing 50 μl of 0.5 M EDTA. Plasma was prepared by centrifugation at 500 × *g* for 20 min and 4 °C, snap-frozen in liquid N_2_ and stored at − 80 °C. The heart was extirpated and separated into left ventricle (LV) and right ventricle, together with lungs and liver, rinsed in saline solution, blotted dry and weighed. A standardized 2 mm slice was taken from the LV using a mouse heart slicer matrix (Zivic Instruments, Pittsburgh, PA, USA). The heart slice was fixated in 4% formalin and embedded in paraffin. Remaining tissue was snap-frozen in liquid nitrogen and stored at − 80 °C^22^.

### Global metabolic profiling

Global biochemical profiles were determined in mouse plasma collected from genotypes/treatment groups as below. Plasma from mice that had been fed HFD or control diet for 52 weeks, were immediately frozen in liquid nitrogen. Samples (4 per treatment group) were shipped to Metabolon Inc. (Durham, NC, USA) https://www.metabolon.com where they were extracted and prepared for analysis using a previously described standard solvent extraction method^69^. Also included were several technical replicate samples created from a homogeneous pool containing a small amount of all study samples. Metabolite profiling was provided by Metabolon Inc. using Ultra High Performance Liquid chromatography/Mass Spectrometry/Mass Spectrometry (UHPLC/MS/MS) and Gas chromatography/Mass Spectrometry (GC/MS). Fractionation and derivisation of samples and detection technologies have been reported previously^70,71^. The analysis yielded a dataset comprising a total of 520 compounds of known identity. Metabolic pathways were visualized using the Cytoscape plugin in the MetaboLync Portal https://portal.metabolon.com. The y-axis of figures is termed “Scaled intensity”, and indicates normalized values in terms of raw area counts. These values are rescaled to set the median equal to 1.

### Complex lipid panel

Complex Lipid Panel identifies up to 1100 individual lipid species (Metabolon Inc.). This platform provides absolute quantitation of 14 lipid classes, including principle phospholipid, sphingolipid and neutral lipid classes. It also provides molecular species concentrations and complete fatty acid composition of each lipid class, thereby offering unparalleled insight into the lipidome. Lipids are extracted from samples in methanol:dichloromethane in the presence of internal standards. The extracts are concentrated under nitrogen and reconstituted in 0.25 ml of 10 mM ammonium acetate methanol: dichloromethane (50:50). The extracts are transferred to inserts and placed in vials for infusion-MS analysis, performed on a Shimazdu LC with nano PEEk tubing and the Sciex SelexIon-5500 QTRAP. The samples are analyzed via both positive and negative mode electrospray. The 5500 QTRAP scan is performed in MRM mode with the total of more than 1100 MRMs. Individual lipid species are quantified by taking the peak area ratios of target compounds and their assigned internal standards, then multiplying by the concentration of internal standard added to the sample. Lipid class concentrations are calculated from the sum of all molecular species within a class, and fatty acid compositions are determined by calculating the proportion of each class comprised by individual fatty acids^72^.

### Measurements of total fatty acid (FA) levels and composition in liver

Lipids were extracted from livers using a mixture of chloroform and methanol. The extracts were trans-esterified using boron trifluoride (BF_3_)-methanol. To remove neutral sterols and non-saponifiable material, extracts of FA methyl esters were heated in 0.5 M potassium hydroxide (KOH) in ethanol–water solution (9:1). Recovered FAs were re-esterified using BF_3_-methanol. The methyl esters were quantified by gas chromatography as previously described^73^.

### Proteomic analysis

LV tissues of four to five biological replicates were used for proteome analysis^74^. The total cell protein was extracted by T-PER Mammalian Protein Extraction Reagent containing Halt Protease and Phosphatase Inhibitor (Thermo Fisher Scientific, Waltham, MA, USA) and homogenized. The samples were transferred to Eppendorf tubes and centrifuged at 14,000 × *g* 10 min 4 °C. The supernatants were then transferred to new Eppendorf tubes, and the protein concentration was determined by Pierce BCA Protein Assay Kit (Thermo Fisher Scientific).

The precipitated proteins were dissolved with 6 M urea in 100 mM ammonium bicarbonate, reduced with dithiothreitol (10 mg/ml) and alkylated with iodoacetamide (25 mg/ml). For total proteome analysis, the proteins were in-solution digested by diluting the urea concentration to 1 M followed by digestion with trypsin overnight at 37 °C. The resulting peptides were desalted and concentrated before mass spectrometry by the STAGE-TIP method using a C18 resin disk (3 M Empore). Each peptide mixture was analyzed by a nEASY-LC coupled to QExactive Plus (Thermo Electron, Bremen, Germany) with EASY Spray PepMap RSLC column (C18, 2 µl, 100 Å, 75 µm × 50 cm). For proteome samples, 120 min LC separation gradient was used. The resulting MS raw files were submitted to the MaxQuant software version 1.6.1.0 for protein identification and label-free quantification (LFQ). Carbamidomethyl (C) was set as a fixed modification and acetyl (protein N-term), carbamyl (N-term) and oxidation (M) were set as variable modifications. First search peptide tolerance of 20 ppm and main search error 4.5 ppm were used. Trypsin without proline restriction enzyme option was used, with two allowed miscleavages. The minimal unique + razor peptides number was set to 1, and the allowed false discovery rate (FDR) was 0.01 (1%) for peptide and protein identification. Label-free quantitation was employed with default settings. The UniProt database with ‘human’ entries (October 2017) was used for the database searches. Known contaminants as provided by MaxQuant and identified in the samples were excluded from further analysis, and Perseus software 1.6.1.3 was used for the statistical analysis of the total proteome MaxQuant results. Significant differentially expressed proteins were analyzed by the use of the principles of Gene Ontology (GO)^75^ that describe gene products in terms of their associated biological process, cellular component or molecular function, and we investigated the use of the protein/enzyme Kyoto Encyclopedia of Genes and Genomes (KEGG) database in the Functional Annotation Chart in the database for annotation, visualization and integrated discovery (DAVID). Heatmaps were generated based on the Z-scores (normalized with mean) of log10-transformed LFQ intensities by pheatmap R package; pheatmap: Pretty Heatmaps. R package version 1.0.12. https://CRAN.R-project.org/package=pheatmap).

### Immunohistochemistry and immunofluorescence

Four micron transverse sections of formalin-fixed, paraffin-embedded mouse hearts and livers were deparaffinized in xylene, rehydrated in alcohol series and immersed in distilled water, followed by high-temperature antigen retrieval in citrate buffer (pH 6) and blocked with 1% bovine serum albumin (Sigma-Aldrich, St. Louis, MO, USA). Slides were stained with primary antibody against mouse anti-ceramide (1:100, Glycobiotech, Kuekels, Germany) and rat anti-mouse antibody against Mac-2 (1:1000, Cedarlane, Burlington, Canada), respectively, for 1 h at room temperature. After washing, slides were incubated for 30 min with biotinylated secondary antibody (goat anti-mouse IgM, Thermo Fisher Scientific) and peroxidase-conjugated secondary antibody (goat anti-rat IgG, Thermo Fisher Scientific), respectively. After washing the slides were incubated in prepared Vectastain ABC kit (Vector Laboratories, Burlingame, CA, USA), rinsed and developed with chromogen for immunoperoxidase staining (DAB Plus, Vector Laboratories). The sections were counterstained with hematoxylin. Omission of the primary antibody was used as negative control^22^. The stained sections were scanned (AxioScan Z1, Carl Zeiss, Oberkochen, Germany), and the amount of positive DAB-staining was quantitatively assessed using z9.uio.no, an in-house analysis application devised for whole slide images, by estimating cross sectional coverage of antibody expression within the tissue relative to the total area of the cross section of the tissue.

All histological analyses were performed blinded of genotype and treatment.

For immunofluorescence, the slides were stained with mouse anti-mouse antibody against ceramide (1:50, Glycobiotech); chicken anti-human antibody against Succinate dehydrogenase complex assembly factor 2, SDHAF2, a mitochondrial complex 2 marker (1:200, LSBio, Seattle, WA, USA); mouse anti-mouse antibody against NLRP3 (1:100, Adipogene, San Diego, CA, USA), rabbit anti-mouse against Caspase-1 p10 (1:100, Santa Cruz Biotechnology, Dallas, TX, USA) and Isolectin IB_4_ Alexa Fluor 568 conjugate, an endothelial cell marker (Molecular Probes, Thermo Fisher Scientific) overnight at 4 °C and counterstained with Alexa Fluor 568 goat anti-mouse IgM; Alexa Fluor 488 goat anti-chicken IgY; Alexa Fluor 488 donkey anti-mouse IgG and Alexa Fluor 488 goat anti-rabbit IgG, respectively. Images were captured using a Zeiss LSM710/Elyra S1 confocal microscope (Jena, Germany) housing a Plan-Apochromat W40x/1.0 DIC M27 objective. Zeiss ZEN Lite software or Adobe Photoshop was used for processing images. Omission of the primary antibody was used as a negative control^76^. The stained sections were scanned (AxioScan Z1, Carl Zeiss), and the amount of positive fluorescent-staining was quantitatively assessed using z9.uio.no, as explained above.

### Microbiota

Cecal content from 25 fasted mice was taken from the cecum with sterile equipment, and immediately snap-frozen in liquid nitrogen and later stored at − 80 °C until DNA extraction (5–8 mice per treatment group). DNA from cecal content was extracted as previously described^77^. In short, samples were resuspended in lysis buffer containing 20 mg/ml lysozyme (Sigma-Aldrich) and incubated at 37 °C for 30 min. Sodium dodecyl sulphate (10%, Sigma-Aldrich) and proteinase K (20 mg/ml, Qiagen, Chatsworth, CA, USA) were then added followed by 30 min incubation at 60 °C. The samples were then homogenized using a bead beater (BioSpec Products, Bartlesville, OK) and 300 mg zirconium beads (0.1 mm; BioSpec Products). Finally, samples were processed with DNeasy mini DNA extraction kit (Qiagen).

DNA libraries were prepared as described elsewhere^78^. Briefly, libraries were generated from polymerase chain reaction (PCR) amplicons targeting the hypervariable regions V3 and V4 of the 16S ribosomal RNA gene, using dual-indexed universal primers 319F (forward) and 806R (reverse) along with Phusion High-Fidelity PCR Master Mix with HF buffer (Thermo Fisher Scientific). Cleaning and normalization of PCR products were performed using the SequalPrep Normalization Plate Kit (Thermo Fisher Scientific). Quality control and quantification of pooled libraries were performed using Agilent Bioanalyzer (Agilent Technologies, Santa Clara, CA, USA) and Kapa Library Quantification Kit (Kapa Biosystems Inc., Wilmington, MA, USA). Sequencing was performed at the Norwegian Sequencing Centre in Oslo, applying the Illumina MiSeq platform and v3 kit (Illumina, San Diego, CA, USA), allowing for 300-base pair paired-end reads. The genera *Shewanella, Pseudomonas,* *Halomonas* and *Cutibacterium* were detected in both negative controls, and were thus treated as contaminants and excluded from the dataset. To control for heterogeneous sequencing depths across the samples, all samples were rarefied to a level of 5789 reads. Calculations of alpha-diversity measures, as well as all subsequent analyses, were performed on this rarefied dataset using QIIME2.

### ELISA, LPS binding protein

Mouse LPS binding protein (LBP) (ELISA kit, HycultBiotech, Weyne, PA, USA) was measured in plasma according to the manufacturer’s protocol.

### Statistical analysis

One-way ANOVA was used to compare differences across the four experimental groups (GraphPad Prism version 8, GraphPad Software, La Jolla, CA, USA). Additionally, linear contrasts within ANOVA for specific comparisons of interest were performed (Welch’s two-sample *t*-test and two-sample *t*-test with pooled estimate of variance) for the metabolomics data. Student’s *t*-tests and Mann–Whitney U tests were conducted to compare the diversity between diet types. Scaled Imp Data: Each biochemical in OrigScale is rescaled to set the median equal to 1. Tables represent fold of change between NLRP3^−/−^ and WT mice on control diet or HFD; and between HFD and control diet in WT or NLRP3^−/−^ mice. Metabolite levels that increase in response to the diet are colored red (*P* ≤ 0.05), and lipid levels that decrease are colored green (*P* ≤ 0.05). Data of metagenomic analysis were analyzed using a least squares linear regression and Wilcoxon-test. Perseus software 1.6.1.3 was used for the statistical analysis of total proteome data. Statistical significance for all data was set at *P* ≤ 0.05. Data are presented as mean ± standard error of the mean (SEM). # representing significant differences between HFD and control diet within one genotype, (i.e. WT or NLRP3^−/−^); and * representing significant differences between the two genotypes (NLRP3^−/−^ and WT) fed either HFD or control diet.

## Supplementary information

Supplementary Figure S1.

Supplementary Figure S2.

Supplementary Figure S3.

Supplementary Figure S4.

Supplementary Figure S5.

Supplementary Legends.
