# Supplementary material for: NLRP3 inflammasome deficiency attenuates metabolic disturbances involving alterations in the gut microbial profile in mice exposed to high fat diet
Source: Sci Rep. 2020 Dec 3;10:21006. doi: 10.1038/s41598-020-76497-1 (PMC7712828; doi:10.1038/s41598-020-76497-1)
Supplement: Supplementary file 1 — Supplementary Figure S1. [file 41598_2020_76497_MOESM1_ESM.docx]

**NLRP3 inflammasome deficiency attenuates metabolic disturbances involving alterations in the gut microbial profile in mice exposed to high fat diet**

Marina Sokolova^1,2^, Kuan Yang^1,2^, Simen H. Hansen^1,2,3^, Mieke C. Louwe^1^, Martin Kummen^1,2,3,4^, Johannes E.R. Hov^1,2,3,5^, Ivar Sjaastad^6,7^, Rolf K. Berge^8^, Bente Halvorsen^1,2^, Pål Aukrust^1,2,8^, Arne Yndestad^1,2^  and Trine Ranheim^1,2*^

^1^Research Institute of Internal Medicine, Oslo University Hospital Rikshospitalet, Oslo Norway; ^2^Institute of Clinical Medicine, Faculty of Medicine, University of Oslo, Oslo Norway; ^3^Norwegian PSC Research Center, Department of Transplantation Medicine, Oslo University Hospital, Oslo, Norway;  ^4^Department of Oncology, Oslo University Hospital Ullevål, Oslo, Norway; ^5^Section of Gastroenterology, Department of Transplantation Medicine, Oslo University Hospital Rikshospitalet, Oslo, Norway; ^6^Institute for Experimental Medical Research, Oslo University Hospital Ullevål, Oslo, Norway; ^7^KG Jebsen Center for Cardiac Research, University of Oslo, Oslo, Norway; ^8^Department of Clinical Science, University of Bergen and Department of Heart Disease, Haukeland University Hospital, Bergen, Norway; ^8^Section of Clinical Immunology and Infectious Diseases, Oslo University Hospital Rikshospitalet, Oslo, Norway.

*Correspondence:

Dr. Trine Ranheim

trine.ranheim@rr-research.no

**Figure S1.**  Food intake and food intake markers. WT and NLRP3^−/−^ male mice were exposed to high fat diet (HFD; 60 cal% fat) or control diet (CD) for 52 weeks. (A) Food intake was assessed at 21 weeks of age by weighing the food and correcting for the amount not eaten, including spillage. [WT: CD, n = 7; HFD, n = 7 and NLRP3^−/−^: CD, n = 7; HFD, n = 7]. (B) Circulating markers of food intake: Gluconate, 2- keto-3-deoxy-gluconate, Stachydrine, and Homostachydrine. No error bars are available for Homostachydrine levels in WT and NLRP3^-/-^ on control diet due to that they were almost below detection limit. [WT: CD, n = 4; HFD, n = 4 and NLRP3^−/−^: CD, n = 4; HFD, n = 4]; each biochemical is rescaled to set the median equal to 1. Data are means ± SEM.

**Figure S2.**  Increased Mac-2 expression in WT mice fed HFD. WT and NLRP3^−/−^ male mice were exposed to high fat diet (HFD; 60 cal% fat) or control diet (CD) for 52 weeks. (A) Representative immunohistochemistry images of Mac-2 staining in liver tissue. Scale bar: 30 µM. (B) Data represent quantification of Mac-2 positive areas in liver tissue relative to total area of liver tissue. *P < 0.017 NLRP3^−/−^ HFD vs. WT HFD. [WT: CD, n = 10; HFD, n = 9 and NLRP3^−/−^: CD, n = 6; HFD, n = 6]. Data are means ± SEM. * representing significant differences between the two genotypes (NLRP3^-/-^ and WT) fed either HFD or control diet.

**Figure S3.** NLRP3 and Caspase-1 protein expression within endothelial cells in left ventricular (LV) tissue. WT male mice were exposed to high fat diet (HFD; 60 cal% fat) or control diet (CD) for 52 weeks. (A) Representative immunofluorescence images of NLRP3 staining in the endothelial cell layer (upper panels) and Caspase-1staining in the endothelial cell layer (lower panels) in LV sections from WT mice. [WT: CD, n = 5; HFD, n = 5]. Scale bar: 20 µM. (B) Quantification of positive cells in the endothelial layer. Data are means ± SEM.

**Figure S4.**  (A) Heatmap presenting differentially expressed proteins in the Fatty acid biosynthesis. (B) Heatmap presenting differentially expressed proteins in the Krebs cycle. Changes in protein abundance (Z-score) are shown. Proteins were grouped according to metabolic pathways. Colors represent either increased (red) or decreased (green) protein abundances. [WT: CD, n = 5 and NLRP3^−/−^: CD, n = 4].

**Figure S5.** HFD increases the Bilophila genus in WT mice. WT and NLRP3^−/−^ male mice were exposed to high fat diet (HFD; 60 cal% fat) or control diet (CD) for 52 weeks. *P=0.04 NLRP3^−/−^ HFD vs. WT HFD. [WT: CD, n = 7; HFD, n = 6 and NLRP3^−/−^: CD, n = 5; HFD, n=6]. Data are means ± SEM. * representing significant differences between the two genotypes (NLRP3^-/-^ and WT) fed either HFD or control diet.
