# Supplementary figures and images for: NLRP3 inflammasome deficiency attenuates metabolic disturbances involving alterations in the gut microbial profile in mice exposed to high fat diet
Source: Sci Rep. 2020 Dec 3;10:21006. doi: 10.1038/s41598-020-76497-1 (PMC7712828; doi:10.1038/s41598-020-76497-1)

**A**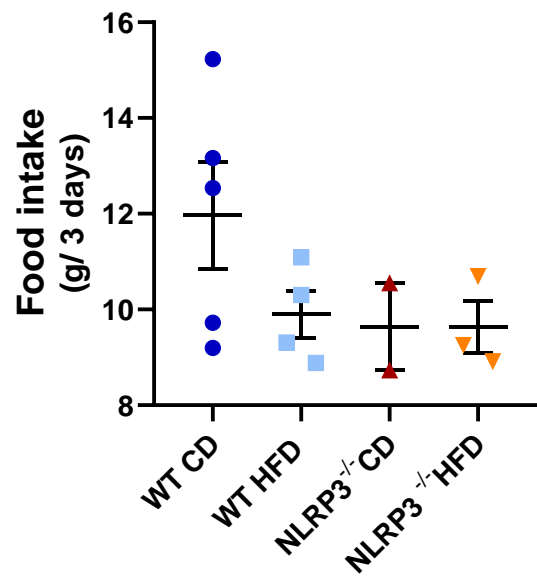**B**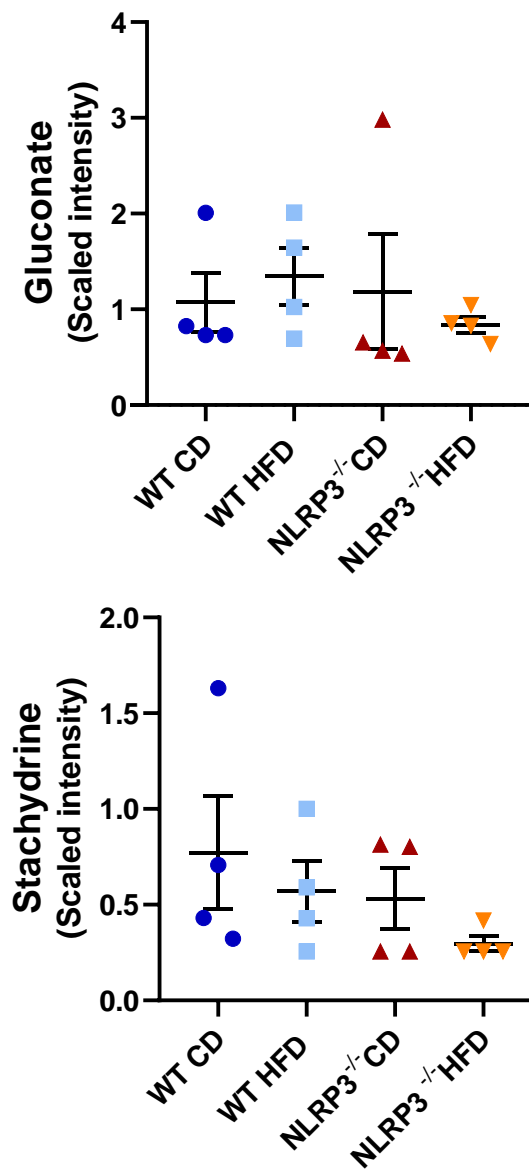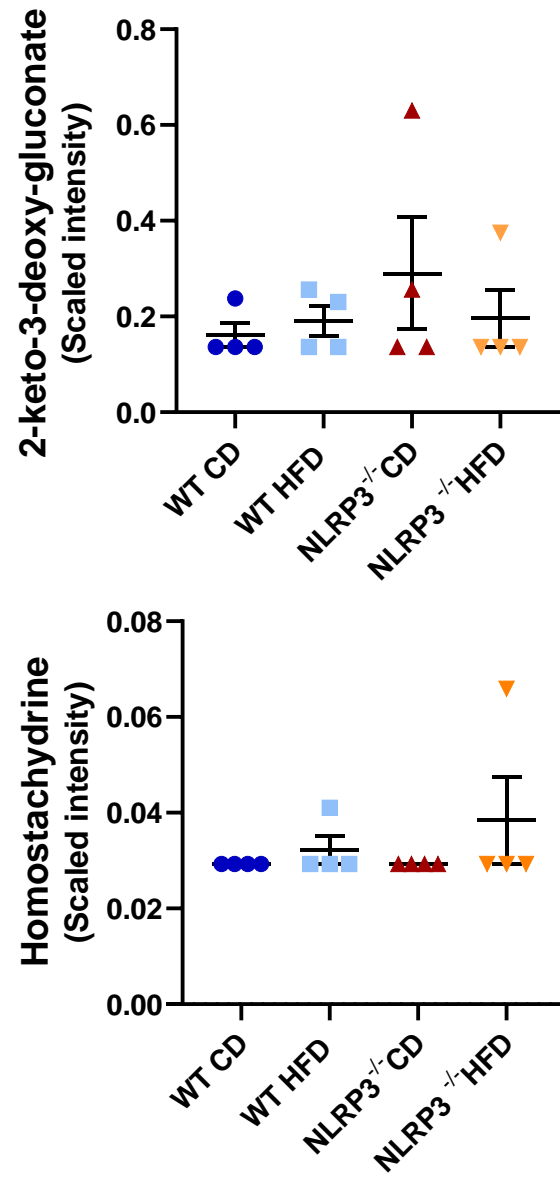

Supplement: Supplementary file 2 — Supplementary Figure S2. [file 41598_2020_76497_MOESM2_ESM.pdf]

**A**

WT CD

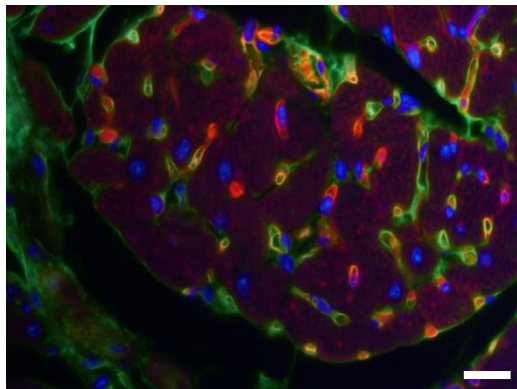

WT HFD

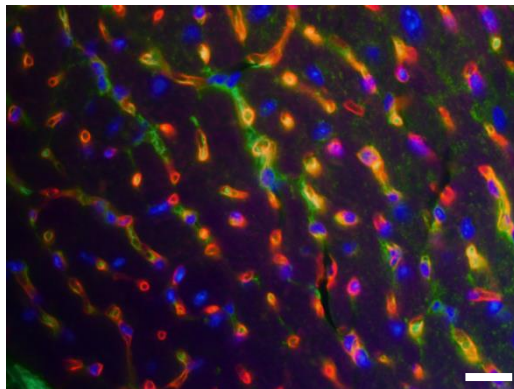

Nuclei NLRP3 Endothelial cells

WT CD

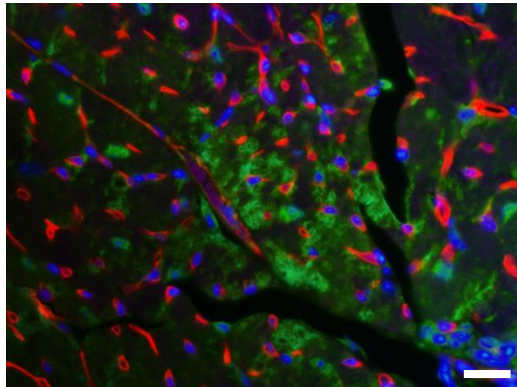

WT HFD

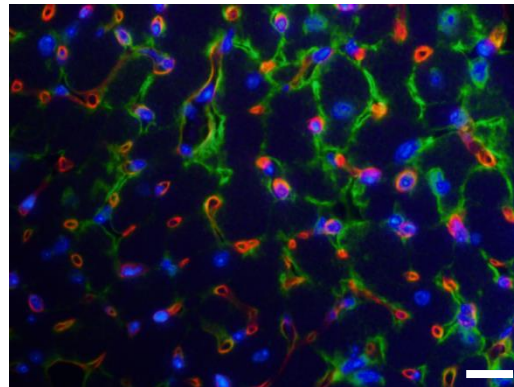

Nuclei Caspase-1 Endothelial cells

**B**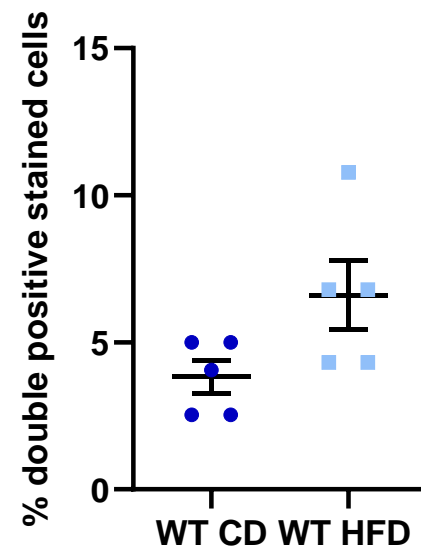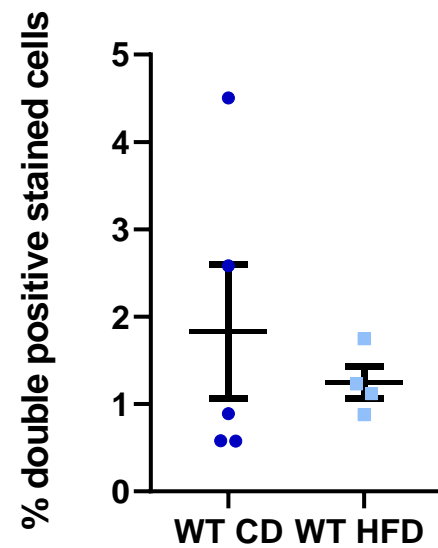

Supplement: Supplementary file 4 — Supplementary Figure S4. [file 41598_2020_76497_MOESM4_ESM.pdf]

**A**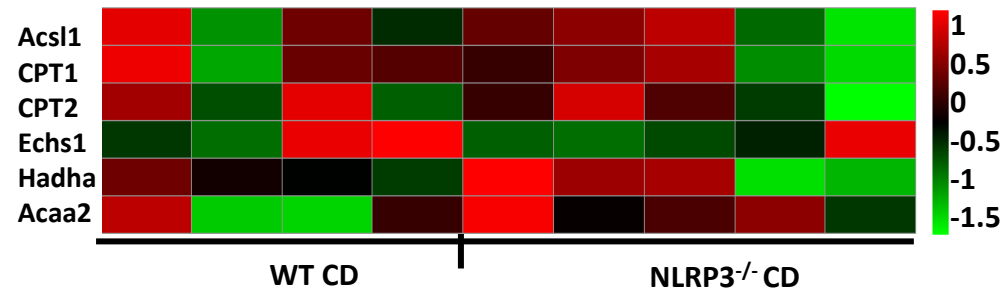**B**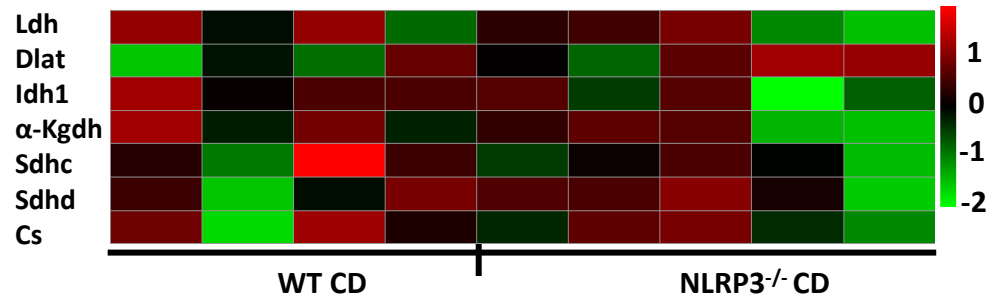

Supplement: Supplementary file 5 — Supplementary Figure S5. [file 41598_2020_76497_MOESM5_ESM.pdf]

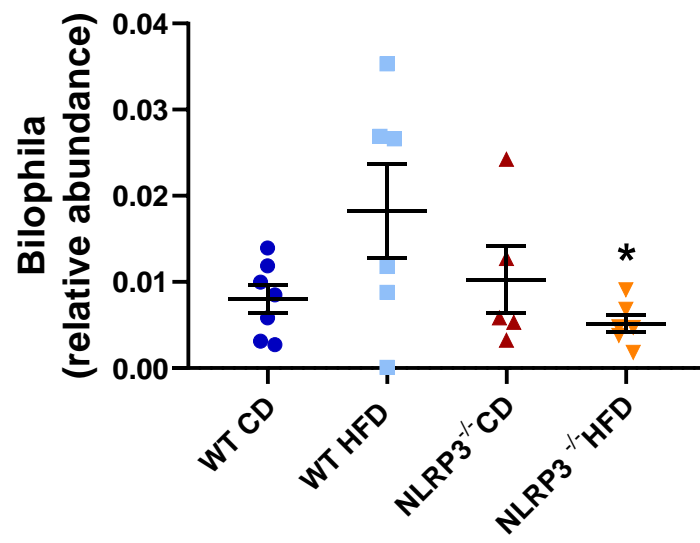

Supplement: Supplementary file 6 — Supplementary Legends. [file 41598_2020_76497_MOESM6_ESM.pdf]
