# Supplementary material for: NLRP3 inflammasome deficiency attenuates metabolic disturbances involving alterations in the gut microbial profile in mice exposed to high fat diet
Source: Sci Rep. 2020 Dec 3;10:21006. doi: 10.1038/s41598-020-76497-1 (PMC7712828; doi:10.1038/s41598-020-76497-1)
Supplement: Supplementary file 3 — Supplementary Figure S3. [file 41598_2020_76497_MOESM3_ESM.pdf]

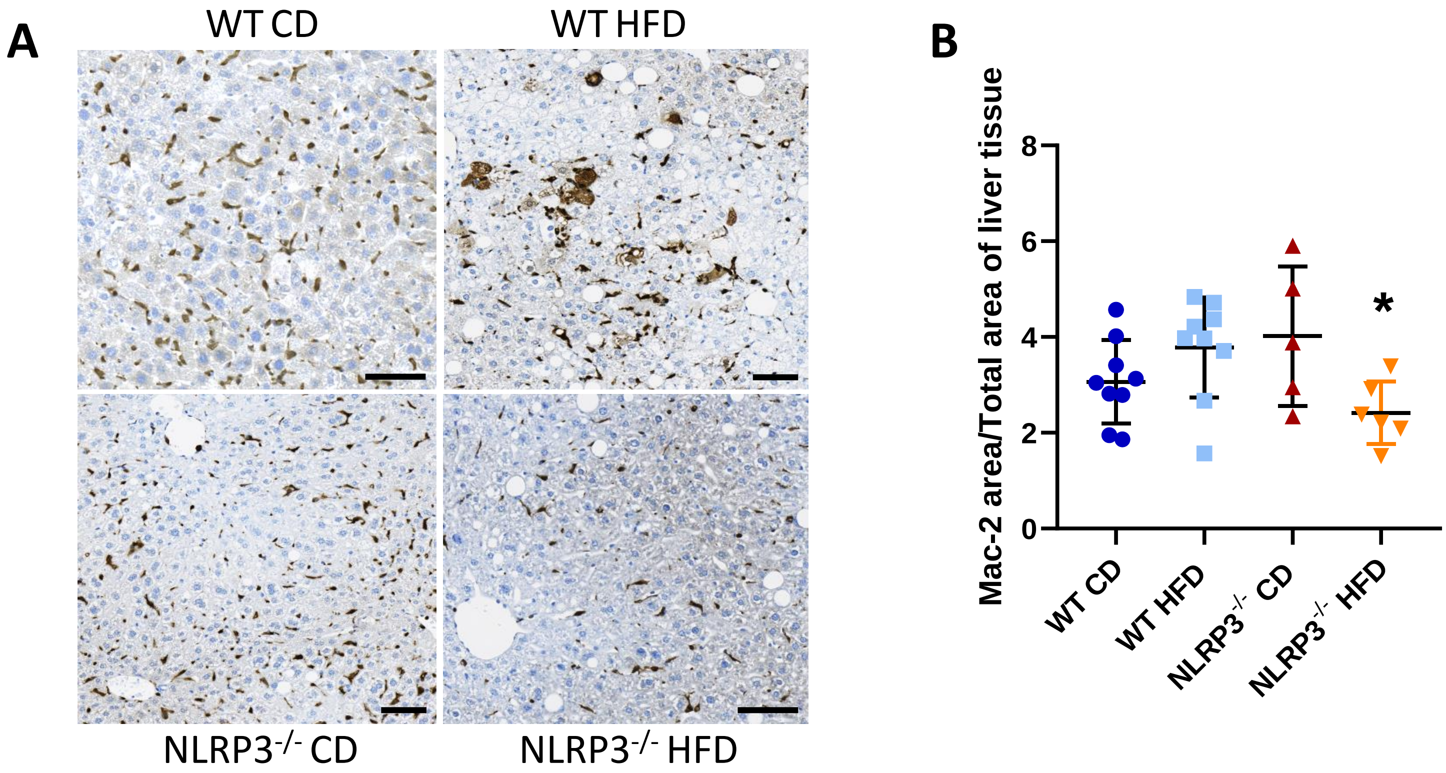

A
WT CD
WT HFD
NLRP3-/- CD
NLRP3-/- HFD
B
Mac-2 area/Total area of liver tissue
8
6
4
2
0
*
WT CD
WT HFD
NLRP3-/- CD
NLRP3-/- HFD
